# Electrophysiological evidence for temporal dynamics associated with attentional processing in the zoom lens paradigm

**DOI:** 10.7717/peerj.4538

**Published:** 2018-04-03

**Authors:** Qing Zhang, Tengfei Liang, Jiafeng Zhang, Xueying Fu, Jianlin Wu

**Affiliations:** 1 Department of Radiology, Affiliated Zhongshan Hospital of Dalian University, Dalian, Liaoning Province, China; 2 Research Center of Brain and Cognitive Neuroscience, Liaoning Normal University, Dalian, Liaoning Province, China

**Keywords:** Visuospatial attention, Zoom lens, Attentional scaling, Event-related potential, N2pc

## Abstract

**Background:**

Visuospatial processing requires wide distribution or narrow focusing of attention to certain regions in space. This mechanism is described by the zoom lens model and predicts an inverse correlation between the efficiency of processing and the size of the attentional scope. Little is known, however, about the exact timing of the effects of attentional scaling on visual searching and whether or not additional processing phases are involved in this process.

**Method:**

Electroencephalographic recordings were made while participants performed a visual search task under different attentional scaling conditions. Two concentric circles of different sizes, presented to the participants at the center of a screen modulated the attentional scopes, and search arrays were distributed in the space areas indicated by these concentric circles. To ensure consistent eccentricity of the search arrays across different conditions, we limited our studies to the neural responses evoked by the search arrays distributed in the overlapping region of different attentional scopes.

**Results:**

Consistent with the prediction of the zoom lens model, our behavioral data showed that reaction times for target discrimination of search arrays decreased and the associated error rates also significantly decreased, with narrowing the attentional scope. Results of the event-related potential analysis showed that the target-elicited amplitude of lateral occipital N1, rather than posterior P1, which reflects the earliest visuospatial attentional processing, was sensitive to changes in the scaling of visuospatial attention, indicating that the modulation of the effect of changes in the spatial scale of attention on visual processing occurred after the delay period of P1. The N1 generator exhibited higher activity as the attentional scope narrowed, reflecting more intensive processing resources within the attentional focus. In contrast to N1, the amplitude of N2pc increased with the expansion of the attentional focus, suggesting that observers might further redistribute attentional resources according to the increased task difficulty.

**Conclusion:**

These findings provide electrophysiological evidence that the neural activity of the N1 generator is the earliest marker of the zoom lens effect of visual spatial attention. Furthermore, evidence from N2pc shows that there is also a redistribution of attentional resources after the action of the zoom lens mechanism, which allows for better perform of the search task in the context of low attentional resolution. On the basis of the timing of P1, N1, and N2pc, our findings provide compelling evidence that visuospatial attention processing in the zoom lens paradigm involves multi-stage dynamic processing.

## Introduction

One of the most notable characteristics of the human attentional system is the limited amount of resources available to it. When performing visual search tasks, the amount of visual information pertinent to a given search situation often exceeds the maximal amount of information that can be processed by the attentional system at any given time. In this case, top-down attention control plays a key role in rationalizing the use of such limited resources for attention processing. Through this mechanism, an observer can choose to process a small number of spatial stimuli while ignoring information associated with other locations. This mechanism was described by the spotlight model, which assumes that attention can be shone over the attended area as a spotlight (Posner & Petersen, 1990). Information in the spotlight is processed efficiently, whereas that outside the spotlight is filtered out. This model is also supported by some neurological evidence. For example, parts of the visual cortex that topologically map to an attended area were shown to exhibit enhanced activity when attention was paid to this area (Tootell et al., 1998; Brefczynski & DeYoe, 1999; Somers et al., 1999).

In realistic situations, however, one may not be able to determine the location of a target, even when the target’s features are known in advance. At this point, a better strategy would be to adjust the scope of attention based on the current situation, aiming to include the possible location of the target. This attentional control mechanism is well captured by the zoom lens model (Eriksen & Yeh, 1985; Eriksen & St. James, 1986). The zoom lens model compares the attentional spotlight to a zoom lens. In this paradigm, the size of the attentional scope can be adjusted according to the top-down setting, resulting in a modification of processing resources distributed over a given area. A typical prediction of the zoom lens model is that a wider spatial distribution of the attentional scope slows processing compared with a more concentrated distribution. This prediction has been confirmed in many studies (Castiello & Umiltà, 1990; Greenwood, Parasuraman & Alexander, 1997; Greenwood & Parasuraman, 1999; Luo, Greenwood & Parasuraman, 2001; Greenwood & Parasuraman, 2004; Song et al., 2006). On the neurophysiological level, modulation of the attention processing resources by the size of the attentional scope seems to be characterized by the neural activity of the visual cortex. For example, Müller et al. (2003) found that in visual search tasks, the level of neural activity in a given retinotopic visual cortex decreased with decreasing the size of the attentional scope. This reflects the notion that the ability to process multiple targets or positions simultaneously is restricted by the limited resource available to the visual cortex (Franconeri, Alvarez & Cavanagh, 2013). However, another interesting issue arises: at what stage of the visuospatial attentional processing the modulation of processing resources takes place?

By recording and analyzing event-related potentials (ERPs) in high temporal resolution, Luo, Greenwood & Parasuraman (2001) investigated the manifestation of the zoom lens paradigm on early visual evoked potentials (P1 and N1) using a “cue-target” paradigm. In their study, the size of the attentional scope was modulated using square cues of different sizes. The cues were divided into three size categories, and search arrays were presented in the space area indicated by the cues. These researchers found that the searching speed of observers accelerated as the search area narrowed. Corresponding to the behavioral performance, the target-induced amplitude of posterior N1 increased with decreasing the search area’s scope, but earlier P1 exhibited the opposite trend. It should be noted that in their study, the number of distractors contained in a search array increased with expanding the search scope. More distractors imply a higher perceptual load, whereas the amplitude of posterior P1 was found to increase with increasing the perceptual load (Handy & Mangun, 2000; Fu et al., 2009). Therefore, in their study, the amplitude of P1 might have been modulated by different perceptual loads rather than different sizes of the attentional scope. In addition, the spatial locations of the cues in their study were also random. Song et al. (2006) argued that these factors might confound the results observed by Luo, Greenwood & Parasuraman (2001). Thus, in the study of Song et al. (2006) the attentional scopes of different sizes were fixed by the three concentric circles presented at the center of a screen. The search arrays were arranged in a circle, randomly distributed within the specified concentric circles. In terms of behavioral results, the study of Song et al. (2006) replicated the results of Luo, Greenwood & Parasuraman (2001). In contrast to Luo, Greenwood & Parasuraman (2001), Song et al. (2006) found that both the amplitudes of P1 and N1 increased with decreasing the scale of attention. It should be pointed out that the experimental design in the study of Song et al. (2006) has some drawbacks that cannot be ignored. In that study, as the scope of attention increased, the eccentricity of the spatial locations at which the search arrays could be distributed also increased. It is known that the spatial resolution, which relates to the ability to discriminate fine patterns in visual stimuli, decreases with increasing eccentricity (Carrasco, 2011). Lower spatial resolution results in more difficult identification of target features. Therefore, in the study of Song et al. (2006) differences between the tasks’ difficulty owing to different eccentricities might confound the size effect of the attentional scope.

In the current study, we further evaluated the multiple processing stages associated with the zoom lens mechanism. Observers were asked to search for a target in searching scopes that had different sizes. Different searching scopes appeared alternately. In this setting, some search arrays would be spread over the overlapping region of two searching scopes. We were only interested in these search arrays, since they belong to different attentional scopes but have perfectly matched physical properties (same eccentricity and number of distractors). Under this scenario, target-elicited neural activity could only be modulated by the different sizes of the attentional scopes, rather than by the physical properties of the search arrays.

In addition, for the first time, we also examined the redistribution of the attentional resources driven by changes in the attentional scopes. The zoom lens model suggests that widening the attentional scope can reduce the density of processing resources in this region, and therefore can increase the difficulty of search tasks (Eriksen & Yeh, 1985; Eriksen & St. James, 1986). However, recent studies confirmed that observers can adjust the allocation of attentional resources according to the current task difficulty (Liu et al., 2016). This means that in the zoom lens paradigm, although expanding the attentional focus increases the search task difficulty, observers may further adjust the allocation of attentional resources so that the features of the target can be accurately identified. The N2pc component is a lateralized negativity at posterior electrode sites (e.g., PO7/PO8) contralateral to the attended item, and is typically triggered at early post-target latencies (∼200 ms). This component is used as a neurological indicator of spatial-based attentional selection (Luck & Hillyard, 1994) and has been confirmed to track the allocation of attentional resources (Töllner et al., 2010; Kiss & Eimer, 2011; Töllner, Conci & Müller, 2015; Liu et al., 2016; Li, Liu & Hu, 2018). In the current study, we used N2pc to test the redistribution of attentional resources driven by changes in the sizes of the attentional scopes.

## Method

### Ethics statement

Data collection conformed to the Declaration of Helsinki and the ethics committee of Zhongshan hospital affiliated to Dalian university approved the research protocol (the approval number: 2017—127). Verbal informed consent was obtained from all the study participants as agreed by the review board.

### Participants

Nineteen paid participants from the Liaoning Normal University community participated in this experiment. One participant was excluded from the analyses owing to poor performance (32% errors). Another two were excluded because of excessive alpha activity. The remaining sixteen participants (average age, 22.9 years; age range, 20–26; seven males; all right-handed) reported normal or corrected-to-normal vision, and had no known neurological or visual disorders.

### Apparatus and stimuli

**Figure 1:**
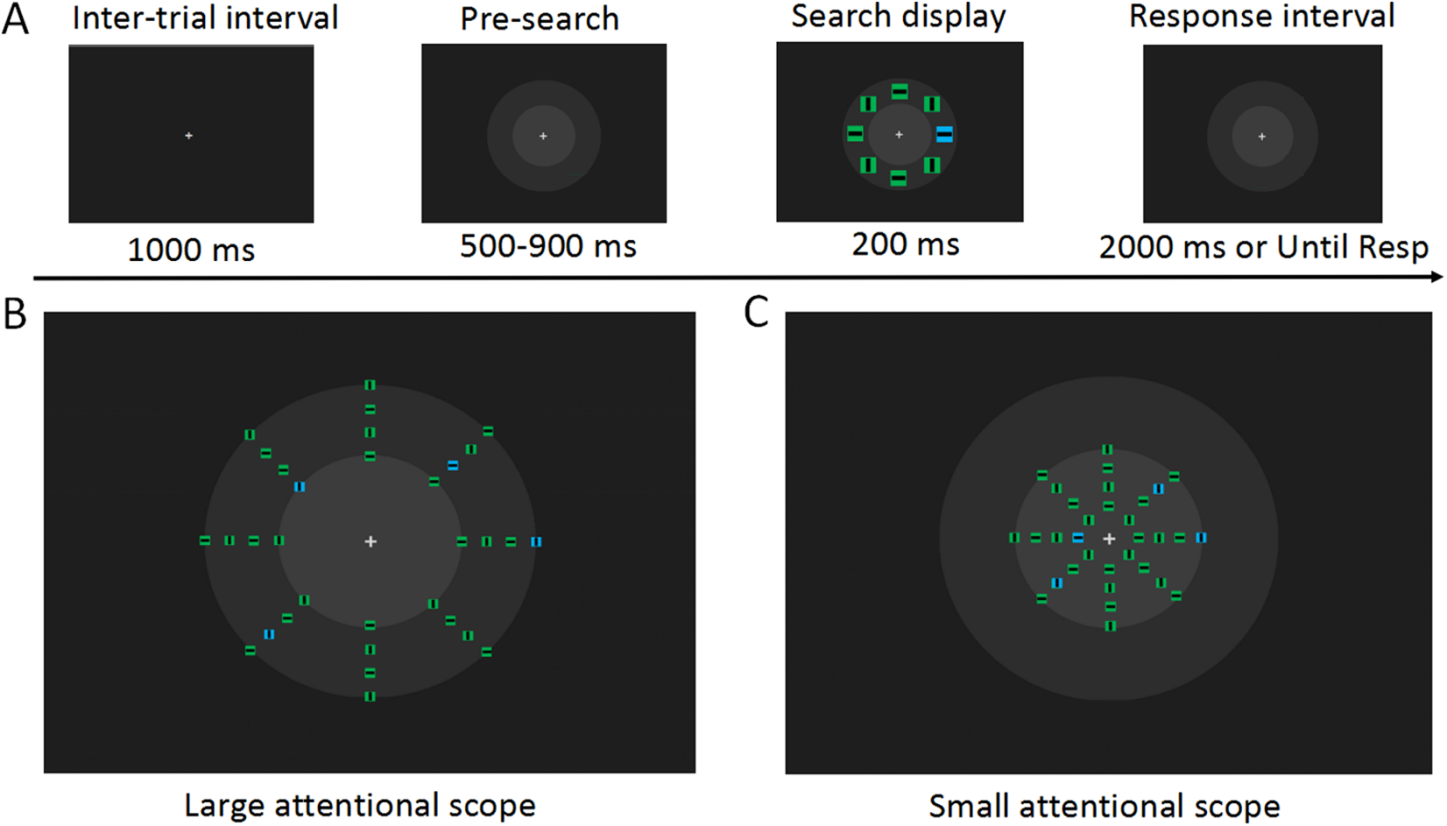
Experimental materials and procedures. (A) Examples of the visual search task (not to scale). At the time 500 ms after the fixation display, concentric circles were presented in front of the search array to prevent the sudden onset of the concentric circles from disturbing the observer on the search task. In the search array, the target stimulus is a blue square in the left visual field. The observer was asked to detect the orientation of the line in this square. In each trial, the search array was distributed in one of the virtual apertures in the larger attentional scope (B) and in the smaller attentional scope (C). Each block contained only one attentional scope, and attentional scopes with different sizes were presented alternately. It can be noticed that the search arrays distributed on the inner circle’s boundary are in the region where the attentional scopes with the two sizes overlap, and these search arrays were of interest to us.

Stimuli were presented on an LCD monitor (refresh rate, 60 Hz, 1,280 × 1,024 resolution) at a viewing distance of 70 cm. In the experiment, a gray central fixation cross (0.26°) always appeared at the center of the monitor (30, 30, 30). The search array was a circular array of eight squares (subtended by 0.65° of the visual angle), arranged equidistantly. There were two different color squares, green (0, 255, 64) and blue (0, 255, 255). In each trial, the color of one square differed from that of the other squares, serving as a target, and the remaining seven squares served as distractors. The color of the target square was consistent within each block and was balanced within the observer. The target square was shown randomly at one of three possible locations on each side of the visual field (Fig. 1). Each square had an embedded horizontal or vertical line (0.25°). The direction of the line was randomly generated. During the pre-stimulation phase, two concentric circles (3.27° and 5.73°, respectively) were presented at the center of the screen, marking the borders of the large and small attentional scopes. The color of the inner circle was light gray (60, 60, 60) and the color of the outer circle was moderate dark gray (45, 45, 45). In the small attentional scope, the search arrays appeared in one of the four virtual rings of 0.82°, 1.64°, 2.46°, or 3.27° within the radius distance from the fixation cross. In the large attentional scope, the search arrays were distributed over one of the four virtual rings of 3.27°, 4.10°, 4.91°, or 5.73°.

### Experimental procedures

Stimuli and procedure were controlled via E-Prime 2.0 routines (Psychology Software Tools, Inc.). At the beginning of each trial, a central fixation cross was presented for 500 ms, followed by the concentric circles, which were displayed for 500–900 ms. Then, a search array was presented for 200 ms. Observers were instructed to respond as quickly and accurately as possible to press the number keys to the right of the keyboard using their right thumb and index finger. When the line within the target square was horizontal, the observers were asked to press the number key “1,” while when a vertical line appeared, the observers were asked to press the number key “2.” The concentric circles disappeared after the observers reacted, or disappeared automatically after 2,000 ms, and were replaced by a solid background, preparing the observers for the next trial.

For each size of the attentional scope, search arrays were randomly distributed over one of the four virtual rings within a limited attentional scope (a detailed description was provided in the previous section). The overlapping region of the two attentional scopes was a virtual ring with a radius of 3.27°. In the current study, we were primarily interested in the search arrays distributed over this overlapping region. These search arrays had exactly the same physical properties (the eccentricity and the number of distractors), while being within the sizes of both attentional scopes. Search arrays that appeared outside of the overlapping region were not included in our analysis owing to their unmatched eccentricities.

The entire experiment contained a total of 24 experimental blocks, with a total of 1,440 test trials. Each block contained 60 test trials, followed by a minimal break of 30 s. Within each block, 24 search arrays were distributed in the overlapping region, and 36 search arrays were distributed in other non-overlapping region. Therefore, for each size of the attentional scope, the number of search arrays distributed in the overlapping region was 288. Each size of the attentional scope lasted six continuous blocks and the order was well-balanced. To make the observers familiar with the task requirements, one training block with 12 trials was offered at the beginning of the experiment. The observers were only allowed to enter the formal experiment when their correct rate exceeded 75%. On the search task, the observers were asked to maintain a central fixation, while minimizing their head and eye movements.

### Electroencephalography recordings and analysis

The ANT Neuro EEGO system was used to record electroencephalographic (EEG) signals using an array of 64 electrodes mounted using a cap with 10/20 montage. Horizontal electrooculograms, which recorded bipolar signals from the outer canthi of the eyes, were used to measure horizontal eye movements. Vertical electrooculogram, which recorded from the FPz site, was used to detect eye blinking. The CPz site was used as the online reference. Electrode impedances were kept below 5 kV with a sampling rate at 500 Hz for on-line recording.

**Figure 2:**
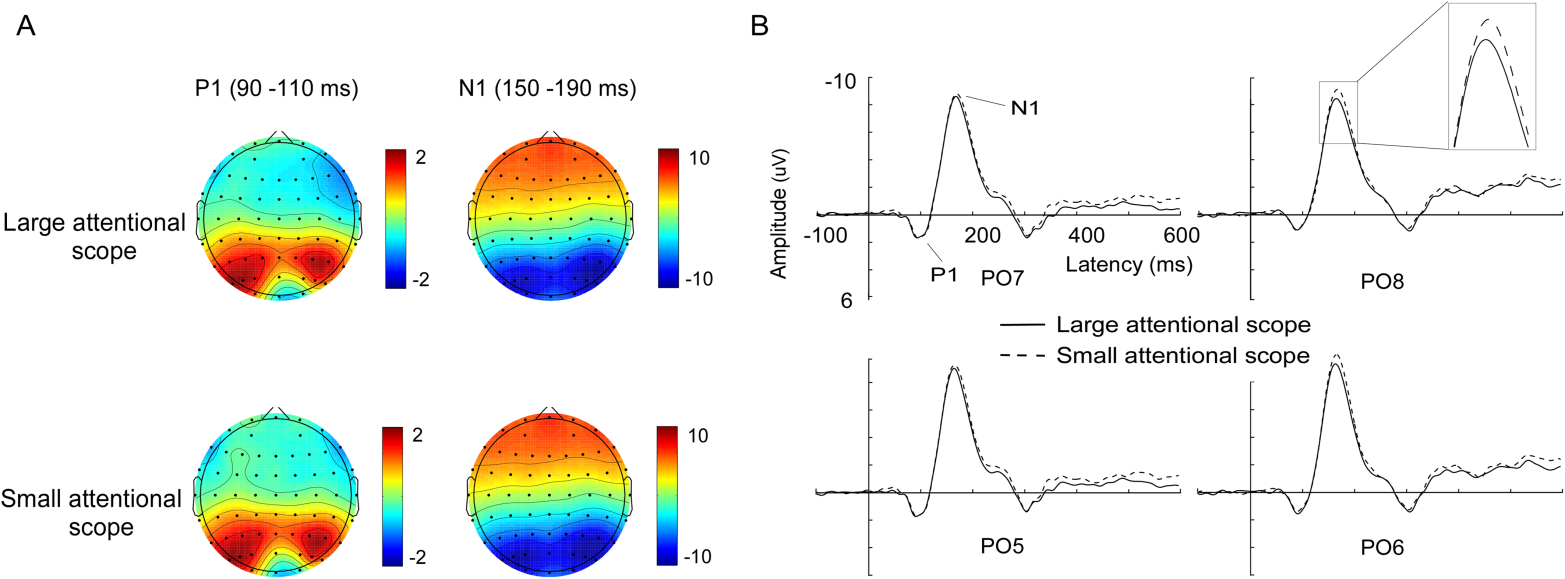
Waveforms and voltage topographies of P1 and N1 components. (A) Voltage topographies of the P1 (90–110 ms) and N1 (150–190 ms) components, for different sizes of attentional scopes. (B) Grand-average ERP (P1 and N1) at the lateral posterior electrode sites (PO5, PO6, PO7, and PO8) evoked by targets (left targets averaged with right targets) for the larger attentional scope (solid lines) and the smaller attentional scope (dashed line). The depicted time epoch (in ms) is marked on the *x*-axis. The stimulus onset time is indexed on the *y*-axis.

Offline signal processing and analysis were performed using the EEGLAB toolbox (Delorme & Makeig, 2004) and MATLAB. Previous studies and our recent work revealed that using the LM reference method weakens the electrical signals from bilateral temporal occipital regions, while using the reference electrode standardization technique (REST) was shown to be promising for improving this situation (Tian & Yao, 2013; Liang et al., 2017), by reconstructing a point far away from all brain sources and the scalp electrodes site (Yao, 2001). Therefore, to restore the electrical signals from posterior regions, the EEG data were re-referenced off-line to the REST references. REST analysis (Yao, 2001) was conducted using the REST software from www.neuro.uestc.edu.cn/rest (Dong et al., 2017). The continuous data were filtered using a high pass filter of 0.10 Hz and a low pass filter of 30 Hz. The EEG data were then divided into segments ranging from 100 ms before to 600 ms after the presentation of the search arrays. The pre-stimulus baseline of 100 ms was used for all analyses. Trials with incorrect behavioral responses, excessive noise or drift (±100 μV), large blinks (±60 μV), and horizontal eye movements (±35 μV) were rejected. Further visual inspection was also performed to confirm an appropriate removal of artifacts and residual saccades. Epochs were then averaged according to the condition and visual hemifield. The N2pc signal in response to the target presentation was quantified at the lateral posterior electrode sites (PO7/8) on the basis of the mean amplitude measured in the 200–300 ms interval after the onset of the search arrays. To investigate the modulation of early visual evoked potentials by the scale effect of the spatial attention, P1 and N1 components over the lateral occipital electrodes (Fig. 2) were calculated. Based on the inspection of grand-average waveforms and previous studies, we chose time windows of 90–110 ms for P1, 150–190 ms for N1, after the search array onset. Given that the laterality effects of the target did not regulate the early visual evoked potentials, the calculation of P1 and N1 was performed by averaging all the trials for each size of the attentional scope.

The Greenhouse–Geisser correction was applied when deemed appropriate. The variance analysis and the *T*-test were analyzed using SPSS Statistics 23 (IBM Corp., Armonk, NY, USA), while the statistical power was executed by G-Power 3.1 (Faul et al., 2007).

## Results

### Results of behavioral tests

Trials with mean reaction time (RT) below or above two standard deviations were excluded (on average, 9.82% trials were excluded). Analysis of RT data revealed faster responses to targets for the smaller attentional scope (424.06 ± 59.15 ms) compared with the larger one (444.82 ± 77.19 ms), (t (15/2) = 2.20, *p* = 0.04, Cohen’s *d* = 0.55). The accuracy of the data also suggested that observers behaved worse for the larger attentional scope (90% ± 2%) compared with the smaller scope scenario (93% ± 1%), (t (15/2) = −2.77, *p* = 0.014, Cohen’s *d* = 0.70). These results were in line with the predictions of the zoom lens model, indicating that as the scope of attention narrowed, the observers were able to identify the feature of the target faster and more accurately.

### ERP results

**P1**. Figure 2 shows the average ERPs (left targets averaged with right targets). In the 90–110 ms time window, P1 was mainly distributed across the bilateral posterior sites. According to the scalp topographies, PO5, PO6, PO7, and PO8 in the bilateral posterior region were selected for further statistical analysis. We submitted P1 peak amplitudes to the analysis of 2 (attentional scopes) × 4 (electrode sites) repeated measures analysis of variance (ANOVA). The results showed that the effects of both the attentional scope (F (1, 15) = 0.18, *p* = 0.681) and the electrode sites (F (3, 45) = 1.70, *p* = 0.21) were not significant. Besides, the interaction between the electrode sites and the attentional scope was also not significant (F (3, 45) = 0.43, *p* = 0.54).

**N1**. As shown in Fig. 2, N1 was also mainly distributed across the bilateral posterior sites and with a later time window (150–190 ms). According to the scalp topographies, PO5, PO6, PO7, and PO8 in the bilateral posterior region were selected for further statistical analysis. Repeated measures ANOVA analysis of 2 (attentional scopes) × 4 (electrode sites) was performed and showed that there was a significant main effect of the attentional scope, (F (1, 15) = 8.53, *p* = 0.01, η_p_^2^ = 0.36). However, the main effect of the electrode sites (F (3, 45) = 0.69, *p* = 0.43) and the interaction between the electrode sites and the attentional scope (F (3, 45) = 3.20, *p* = 0.09) were not significant. Further examination of the main effects of the attentional scope showed that the targets for the smaller attentional scope (−8.43 ± 2.26 μV) elicited a more negative N1 compared with the larger attentional scope (−7.95 ± 2.48 μV), (t (15) = −2.921, *p* = 0.01, Cohen’s *d* = 0.73), indicating that as the size of the attentional scope decreased, the neural generators of P1 became more active.

**Figure 3:**
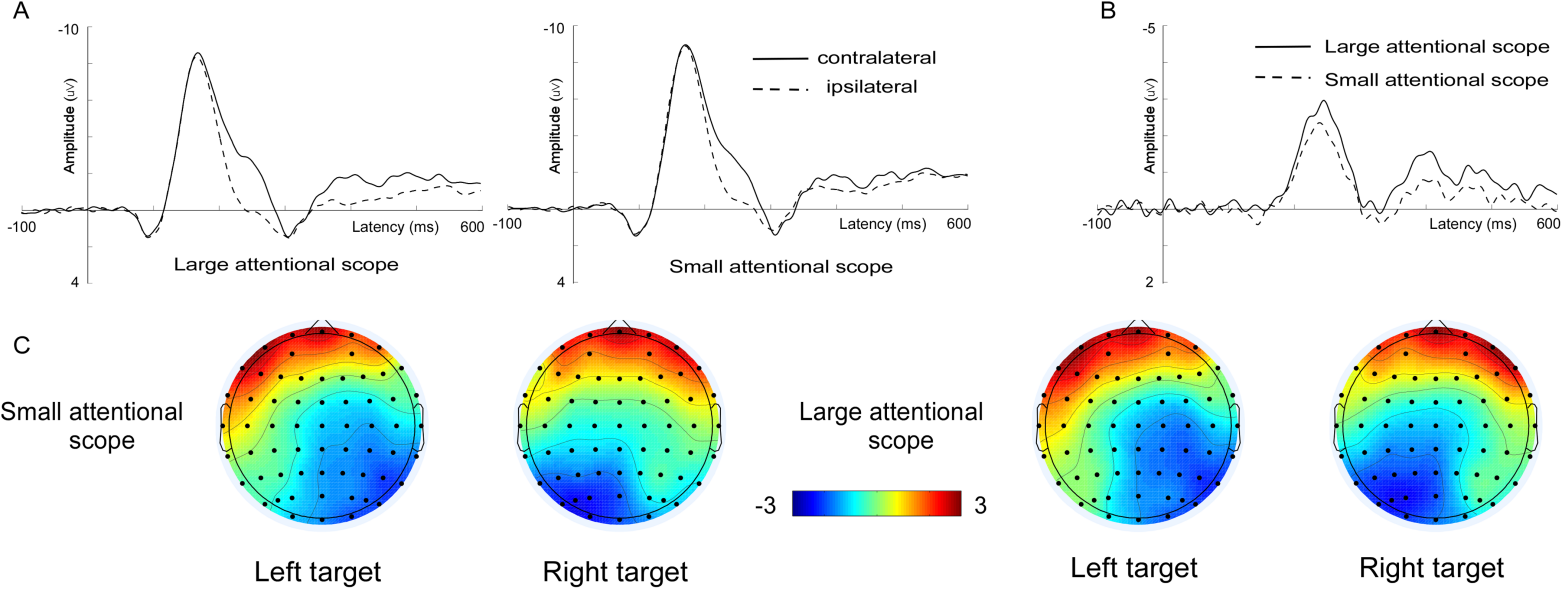
Waveforms and voltage topographies of N2pc components. (A) Grand-average ERPs evoked by targets at the contra- (solid lines) and ipsilateral (dashed line) electrode sites (PO7/8). (B) The resulting N2pc difference wave. N2pc components are shown separately for the larger attentional scope (solid lines) and the smaller attentional scope (dashed line). (C) Topography of the amplitude of the grand-average ERPs from 200 to 300 ms after onset of search array for different sizes of attentional scopes. The depicted time epoch (in ms) is marked on the *x*-axis. The search array onset is indexed on the *y*-axis.

**N2pc**. Grand-average ERPs at the electrode sites PO7/PO8 as well as N2pc difference waves are shown in Fig. 3. Analysis of the mean amplitude (200–300 ms) of N2pc showed that N2pc was more pronounced for the larger attentional scope (−1.96 ± 0.93 μV) compared with the smaller attentional scope (−1.48 ± 0.81 μV), (t (15) = −3.25, *p* = 0.005, Cohen’s *d* = 0.82).

## Discussion

The present study investigated the different stages of attentional processing in the zoom lens paradigm. More specifically, we attempted to examine the effects of changes in the attentional scope on early visual evoked potentials (P1/N1) and N2pc, reflecting the redistribution of attentional resources. In agreement with previous studies (Castiello & Umiltà, 1990; Greenwood, Parasuraman & Alexander, 1997; Greenwood & Parasuraman, 1999; Luo, Greenwood & Parasuraman, 2001; Greenwood & Parasuraman, 2004; Song et al., 2006), during search tasks within the smaller attentional scope, the observers’ search speed and accuracy were significantly better compared with those on search tasks within the larger attentional scope. Importantly, the search arrays of interest had exactly the same physical properties (e.g., the same number of distractors and eccentricity) because they were distributed over the overlapping region of the attentional scopes of both sizes (small and large). In this case, the enhanced search efficiency could only be modulated by the scale effect of visual spatial attention, as the zoom lens model pointed out. Our ERP results showed that changes in the attentional scope modulated the target-elicited P1, N1, and N2pc differently, indicating that visual attention processing in the zoom lens paradigm is characterized by multi-stage dynamic processing.

The P1 component originating in the extrastriate cortex represents the earliest stage in the visual processing modulated by spatial attention. In the current study, we found that the amplitude of P1 did not change with the scaling of spatial attention, suggesting that the scale effect of visual spatial attention does not modulate the exogenous-like P1 component. Unlike the current results, previous studies have found that the amplitude of P1 was modulated by different sizes of attentional scopes. Luo, Greenwood & Parasuraman (2001) found that the target-induced amplitude of lateral occipital P1 decreased as the scaling of spatial attention shrunk, by adjusting the size of the attentional scope by presenting square cues at different spatial locations. However, when used a concentric circle centered at the fixation to mark the scaling of spatial attention, Song et al. (2006) showed that the amplitude of P1 increased with decreasing size of attentional scope. These inconsistent results may arise from the differences in experimental design between their studies, as pointed out in detail in the Introduction section. Therefore, when we adopt a more rigorous experimental design (such as completely matching the physical properties of the search array under different sizes of attentional scopes, under this setting, the amplitude of P1 can only be modulated by the scaling of spatial attention), failing to duplicate their results. It is generally believed that the neural activity of the P1 generator is related to the “sensory gain control” mechanism (Mangun & Hillyard, 1991; Eimer, 1993), reflecting enhanced perceptual processing of relevant spatial locations. As many studies have found, the amplitude of lateral occipital P1 can be modulated by cued spatial attention (Mangun & Hillyard, 1991; Heinze et al., 1994; Clark, Fan & Hillyard, 1995; Clark & Hillyard, 1996; Fu et al., 2005). From this point of view, the neural activity of the P1 generator might only be sensitive to the presence or absence of the spotlight illumination of attention, but not to the intensity of the illumination (i.e., the density of processing resources assuming the zoom lens mechanism), since the search arrays for all conditions were distributed under the attentional spotlight.

Different from P1, we found that the lateral occipital component N1 with a temporal lag could be sensitive to changes in the size of the attentional scope. Specifically, the amplitude of N1 increased with decreasing the size of the attentional scope. This is consistent with the findings of Luo, Greenwood & Parasuraman (2001). By enforcing a more rigorous experimental control, we further confirmed the relationship between the amplitude of the N1 component and the zoom lens mechanism. The zoom lens model indicated that the processing resources distributed in a given area increase with decreasing the size of the attentional scope (Eriksen & St. James, 1986). This view suggests that the neural activity of the N1 generator may be closely related to processing resources that vary with the size of the attentional focus. Luo, Greenwood & Parasuraman (2001) argued that this property of N1 may reflect a gradient of attention allocation. In their view, as the size of the attentional scope decreases, the spatial gradient of attention is narrowed, leading to a greater activation of the N1 generator. However, they did not answer the key question whether the neuronal activity of the N1 generator is involved in the zooming out and zooming in of the attentional spotlight.

According to the zoom lens model, it is unlikely that the scale effect of visual spatial attention is driven by bottom-up factors; rather, it should be the result of a top-down modulation. Some recent studies have explored the involvement of higher brain regions in flexible modulation of the scale effect of spatial attention. Chen et al. (2009) observed that the right temporal parietal cortex could simultaneously participate in the voluntary adjustment of the zoom-in and zoom-out of spatial attention. Subsequently, Ronconi et al. (2014) further found that transcranial magnetic stimulation, applied to the right frontal eye fields but not to the left frontal eye fields, interfered with the attentional mechanism of magnification and demagnification of attentional focus. Therefore, it is reasonable to assume that the observed effect of N1 in the current study might be modulated by higher-level control regions, such as the right frontal parietal network. This view is consistent with the findings of Knight (1997) who revealed that specific lesions of the frontal and parietal regions could affect the activity intensity of the N1 generator in the lateral occipital cortex. Future work will be needed to explore the relationship between posterior N1 and the right frontal parietal network.

In addition, we also explored the modulation of the late N2pc components by the scale effect of the attentional scope. We found that, contrary to the earlier occurring N1, the amplitude of N2pc increased with increasing the scale of spatial attention. N2pc is generally used as a neurological indicator of attentional spatial selection (Luck & Hillyard, 1994), and its amplitude is determined by both bottom-up and top-down factors. For example, it has been shown that more salient target features could elicit a higher amplitude of N2pc (Töllner et al., 2011; Zhao et al., 2011). In the current study, however, the amplitude of N2pc was less likely to be driven by the bottom-up salience of target features. The search arrays for the both attentional scopes had exactly the same physical properties. In this case, the conspicuousness of the salience of the target features could only be modulated by the size of the attentional scope. The zoom lens model assumes that processing resources decrease with increasing the focus of attention (Eriksen & St. James, 1986). It has been suggested that when processing resources are abundant, it is easier to attract attention by salient singleton (Lavie & Tsal, 1994; Lavie, 1995). This means that a small attentional scope should increase the amplitude of N2pc, as opposed to the currently observed pattern. Therefore, the amplitude of N2pc is more likely to be modulated by the redistribution of attentional resources.

It has been demonstrated that the amplitude of N2pc is also sensitive to the redistribution of attentional resources that is modulated by the task difficulty (Töllner, Conci & Müller, 2015; Liu et al., 2016). In the current study, as the size of the attentional scope increased, the search efficiency became worse. In this case, observers might need to work harder in order to better recognize the feature of the target. As a result, more attentional resources would be needed in the zooming out of the attentional focus, resulting in a higher amplitude of N2pc. Thus, while the amplitude of N2pc might be jointly determined by both singleton salience and redistribution of the attentional resources, the latter contributed more to the pattern observed in the current study.

It was shown previously that top-down modulation associated with the right frontal parietal network forms the neural basis of the zoom lens mechanism. In light of the above discussion, this modulation was likely to occur before the redistribution of attentional resources reflected by N2pc, approximately within the N1 delay period after the search arrays were presented. Based on this evidence, we speculated that the scaling effect of spatial attention during visual search may operate in at least three different stages. The first stage is the earliest visual spatial attention processing. At this stage, search arrays for different sizes of the attentional scope are firstly perceived with no difference. In the subsequent stages, the spatial gradient of the attention system is modulated by the zoom lens mechanism, as indicated by changes in the activity of the N1 generator. Before the final behavioral response, there is a redistribution of attentional resources. This process is reflected in the modulation of N2pc and is probably driven by reduced attentional resolution as the size of the attentional scope increases.

## Conclusion

In summary, utilizing high temporal-resolution ERP technology, we provided solid evidence to support the view that the modulation of the zoom lens effect on visual processing during search tasks involves multiple dynamic processing stages. Our results suggest that lateral occipital N1 might be the neurophysiological marker of the attentional scaling effect during visual search. In addition, there was also a redistribution of attentional resources after the action of the zoom lens mechanism, indicating that top-down control mechanisms are involved in the post-attentional scaling process.

## Supplemental Information

10.7717/peerj.4538/supp-1 Supplemental Information 1 Raw data.

## Additional Information and Declarations

### Data Availability

The following information was supplied regarding data availability: The raw data has been supplied as Supplemental Dataset Files.

## References

[ref-1] Brefczynski JA, DeYoe EA (1999). A physiological correlate of the ‘spotlight’ of visual attention. Nature Neuroscience.

[ref-2] Carrasco M (2011). Visual attention: the past 25 years. Vision Research.

[ref-3] Castiello U, Umiltà C (1990). Size of the attentional focus and efficiency of processing. Acta Psychologica.

[ref-4] Chen Q, Marshall JC, Weidner R, Fink GR (2009). Zooming in and zooming out of the attentional focus: an fMRI study. Cerebral Cortex.

[ref-5] Clark VP, Fan S, Hillyard SA (1995). Identification of early visual evoked potential generators by retinotopic and topographic analyses. Human Brain Mapping.

[ref-6] Clark VP, Hillyard SA (1996). Spatial selective attention affects early extrastriate but not striate components of the visual evoked potential. Journal of Cognitive Neuroscience.

[ref-7] Delorme A, Makeig S (2004). EEGLAB: an open source toolbox for analysis of single-trial EEG dynamics including independent component analysis. Journal of Neuroscience Methods.

[ref-8] Dong L, Li F, Liu Q, Wen X, Lai Y, Xu P, Yao D (2017). MATLAB toolboxes for reference electrode standardization technique (REST) of scalp EEG. Frontiers in Neuroscience.

[ref-9] Eimer M (1993). Spatial cueing, sensory gating, and selective response preparation: an ERP study on visuo-spatial orienting. Electroencephalography and Clinical Neurophysiology.

[ref-10] Eriksen CW, St. James JD (1986). Visual attention within and around the field of focal attention: a zoom lens model. Perception & Psychophysics.

[ref-11] Eriksen CW, Yeh YY (1985). Allocation of attention in the visual field. Journal of Experimental Psychology: Human Perception and Performance.

[ref-12] Faul F, Erdfelder E, Lang AG, Buchner A (2007). G*Power 3: a flexible statistical power analysis program for the social, behavioral, and biomedical sciences. Behavior Research Methods.

[ref-13] Franconeri SL, Alvarez GA, Cavanagh P (2013). Flexible cognitive resources: competitive content maps for attention and memory. Trends in Cognitive Sciences.

[ref-14] Fu S, Caggiano DM, Greenwood PM, Parasuraman R (2005). Event-related potentials reveal dissociable mechanisms for orienting and focusing visuospatial attention. Cognitive Brain Research.

[ref-15] Fu S, Huang Y, Luo Y, Wang Y, Fedota J, Greenwood PM, Parasuraman R (2009). Perceptual load interacts with involuntary attention at early processing stages: event-related potential studies. NeuroImage.

[ref-16] Greenwood PM, Parasuraman R (1999). Scale of attentional focus in visual search. Perception & Psychophysics.

[ref-17] Greenwood PM, Parasuraman R (2004). The scaling of spatial attention in visual search and its modification in healthy aging. Perception & Psychophysics.

[ref-18] Greenwood PM, Parasuraman R, Alexander GE (1997). Controlling the focus of spatial attention during visual search: effects of advanced aging and Alzheimer disease. Neuropsychology.

[ref-19] Handy TC, Mangun GR (2000). Attention and spatial selection: electrophysiological evidence for modulation by perceptual load. Perception & Psychophysics.

[ref-20] Heinze HJ, Mangun GR, Burchert W, Hinrichs H, Scholz M, Münte TF, Gös A, Scherg M, Johannes S, Hundeshagen H, Gazzaniga MS, Hillyard SA (1994). Combined spatial and temporal imaging of brain activity during visual selective attention in humans. Nature.

[ref-21] Kiss M, Eimer M (2011). Attentional capture by size singletons is determined by top-down search goals. Psychophysiology.

[ref-22] Knight R (1997). Distributed cortical network for visual attention. Journal of Cognitive Neuroscience.

[ref-23] Lavie N (1995). Perceptual load as a necessary condition for selective attention. Journal of Experimental Psychology Human Perception and Performance.

[ref-24] Lavie N, Tsal Y (1994). Perceptual load as a major determinant of the locus of selection in visual attention. Perception & Psychophysics.

[ref-25] Li C, Liu Q, Hu Z (2018). Further evidence that N2pc reflects target enhancement rather than distracter suppression. Frontiers in Psychology.

[ref-26] Liang T, Hu Z, Li Y, Ye C, Liu Q (2017). Electrophysiological correlates of change detection during delayed matching task: a comparison of different references. Frontiers in Neuroscience.

[ref-27] Liu Q, Lin S, Zhao G, Roberson D (2016). The effect of modulating top-down attention deployment on the N2pc/PCN. Biological Psychology.

[ref-28] Luck SJ, Hillyard SA (1994). Spatial filtering during visual search: evidence from human electrophysiology. Journal of Experimental Psychology: Human Perception and Performance.

[ref-29] Luo YJ, Greenwood PM, Parasuraman R (2001). Dynamics of the spatial scale of visual attention revealed by brain event-related potentials. Cognitive Brain Research.

[ref-30] Mangun GR, Hillyard SA (1991). Modulations of sensory-evoked brain potentials indicate changes in perceptual processing during visual-spatial priming. Journal of Experimental Psychology Human Perception and Performance.

[ref-31] Müller NG, Bartelt OA, Donner TH, Villringer A, Brandt SA (2003). A physiological correlate of the “zoom lens” of visual attention. Journal of Neuroscience.

[ref-32] Posner MI, Petersen SE (1990). The attention system of the human brain. Annual Review of Neuroscience.

[ref-33] Ronconi L, Basso D, Gori S, Facoetti A (2014). TMS on right frontal eye fields induces an inflexible focus of attention. Cerebral Cortex.

[ref-34] Somers DC, Dale AM, Seiffert AE, Tootell RB (1999). Functional MRI reveals spatially specific attention modulation in human primary visual cortex. Proceedings of the National Academy of Sciences of the United States of America.

[ref-35] Song WQ, Li X, Luo YJ, Du BQ, Ji XM (2006). Brain dynamic mechanisms of scale effect in visual spatial attention. NeuroReport.

[ref-36] Tian Y, Yao D (2013). Why do we need to use a zero reference? Reference influences on the ERPs of audiovisual effects. Psychophysiology.

[ref-37] Töllner T, Conci M, Müller HJ (2015). Predictive distractor context facilitates attentional selection of high, but not intermediate and low, salience targets. Human Brain Mapping.

[ref-38] Töllner T, Zehetleitner M, Gramann K, Müller HJ (2010). Top-down weighting of visual dimensions: behavioral and electrophysiological evidence. Vision Research.

[ref-39] Töllner T, Zehetleitner M, Gramann K, Müller HJ (2011). Stimulus saliency modulates pre-attentive processing speed in human visual cortex. PLOS ONE.

[ref-40] Tootell RB, Hadjikhani N, Hall EK, Marrett S, Vanduffel W, Vaughan JT, Dale AM (1998). The retinotopy of visual spatial attention. Neuron.

[ref-41] Yao D (2001). A method to standardize a reference of scalp EEG recordings to a point at infinity. Physiological Measurement.

[ref-42] Zhao G, Liu Q, Zhang Y, Jiao J, Zhang Q, Sun H, Li H (2011). The amplitude of N2pc reflects the physical disparity between target item and distracters. Neuroscience Letters.

